# Correlation between Macular Thickness and Visual Field in Early Open Angle Glaucoma: A Cross-Sectional Study

**Published:** 2017

**Authors:** Behzad FALLAHI MOTLAGH, Ali SADEGHI

**Affiliations:** 1 Ophthalmology Department, Tabriz University of Medical Science, Tabriz, Iran

**Keywords:** Optical Coherence Tomography, Visual Fields, Glaucoma, Macular Thickness

## Abstract

The aim of this study was to correlate macular thickness and visual field parameters in early glaucoma. A total of 104 eyes affected with early glaucoma were examined in a cross-sectional, prospective study. Visual field testing using both standard automated perimetry (SAP) and shortwave automated perimetry (SWAP) was performed. Global visual field parameters, including mean deviation (MD) and pattern standard deviation (PSD), were recorded and correlated with spectral domain optical coherence tomography (SD-OCT)-measured macular thickness and asymmetry. Average macular thickness correlated significantly with all measures of visual field including MD-SWAP (r = 0.42), MD-SAP (r = 0.41), PSD-SWAP (r = -0.23), and PSD-SAP (r = -0.21), with P-values <0.001 for all correlations. The mean MD scores (using both SWAP and SAP) were significantly higher in the eyes with thin than in those with intermediate average macular thickness. Intraeye (superior macula thickness – inferior macula thickness) asymmetries correlated significantly with both PSD-SWAP (r = 0.63, P < 0.001) and PSD-SAP (r = 0.26, P = 0.01) scores. This study revealed a significant correlation between macular thickness and visual field parameters in early glaucoma. The results of this study should make macular thickness measurements even more meaningful to glaucoma specialists.

## INTRODUCTION

Although ophthalmoscopy, optic nerve imaging, and perimetry have been traditionally employed to aid in the diagnosis and surveillance of glaucoma, these techniques may be insufficient in early diagnosis of the disease. Many glaucoma experts agree that significant retinal ganglion cell (RGC) damage can occur before standard tests detect a functional loss in vision [1]. Previous studies have shown that a considerable number of RGCs can be lost before any defect in the standard automated perimetry [2-4]. At the moment, optical coherence tomography (OCT) has become a valuable instrument for monitoring glaucomatous structural changes [5]. With the advent of spectral domain OCT (SD-OCT) and its enhanced axial resolution and scan speed, the capability of OCT to assess macular thickness increased even further. In addition, macular thickness measurement on SD-OCT is highly reproducible, with low intravisit and intervisit variations [6-8]. This high reproducibility and less variation may help in easier detection of glaucomatous progression. The relationship between structural changes such as the macular RGC complex thickness and functional outcomes assessed by visual field analysis in patients with glaucoma is an interesting topic of study in the current literature [9-13]. In fact, some previous studies have shown significant correlations between macular thickness loss and Humphrey Visual Field (HVF) parameters in both glaucomatous and normal eyes [14-16]. The available reports, however, are scarce and still conflicting in this regard [17-22]. Therefore, this study aimed to examine a possible correlation between SD-OCT-measured macular thickness, as a measure of structure, and visual field parameters, as markers of visual function, in Iranian patients with glaucoma.

## MATERIALS AND METHODS

A total of 104 eyes diagnosed with glaucoma were examined in this prospective, cross-sectional study conducted in a tertiary eye hospital between 2014 and 2016. The ethics committee of the Tabriz University of Medical Sciences approved this study and informed consent was provided by all subjects. Functional and structural defects, as defined by Hodapp, Parrish, and Anderson [23], were used for the diagnosis of glaucoma. Briefly, the criteria for diagnosing glaucomatous damage were as follows: a Glaucoma Hemifield Test outside normal limits on at least two fields; or a cluster of three or more non-edge points in a location typical for glaucoma, all of which are depressed on the pattern deviation plot at a level of P < 5% and one of which is depressed at a level of P < 1% on two consecutive fields; or a corrected pattern standard deviation that occurs in less than 5% of normal fields on two consecutive fields. The inclusion criteria were as follows: best-corrected visual acuity greater than 5/10; spherical equivalent of refractive error under ±5 diopters; cup-to-disc ratio greater than 0.5 or an intereye asymmetry of cup-to-disc ratio greater than 0.2; and documentation of early glaucoma stage according to the Hodapp, Parrish, and Anderson staging system [23]). The exclusion criteria were as follows: any evidence of retinal pathology that could influence retinal thickness analysis (diabetic retinopathy, senile retinal degeneration, epiretinal membrane, any retinal vascular accidents, or uveitis and its complications); any cornea, lens, or vitreous cavity opacity that could influence image quality; refractive error greater than ± 5 diopters; a history of oral intake of corticosteroid or immunosuppressive agents within the previous 6 months; a history of cataract or any other intraocular surgery; best-corrected visual acuity less than 5/10; failure to record SD-OCT algorithm; and signal strengths of SD-OCT under 15 dB. Complete ophthalmologic examinations comprising determination of the best-corrected visual acuity and refraction, tonometry (using Goldman tonometer), slit-lamp examination using a 90D Volk lens (including determination of the cup-to-disc ratio), gonioscopy, fundus examination with dilated pupil, and pachymetry were carried out in all eyes. Visual field testing including both standard automated perimetry (SAP) and shortwave automated perimetry (SWAP) with 30-2 protocol was performed using a standard Humphrey visual field analyzer (Carl Zeiss Meditec Inc., Dublin, CA). Global visual field parameters including mean deviation (MD) and pattern standard deviation (PSD) were recorded. All eyes underwent macular thickness measurement and asymmetry analysis using SPECTRALIS® SD-OCT software (Heidelberg Engineering GmbH, Heidelberg, Germany). For this purpose, retinal thickness along 61 lines in the central 20 degrees of each eye was measured. The average macular thickness (AMT) as well as the average macular thickness for superior-half and inferior-half portions were calculated and displayed for each eye. Finally, macular thickness parameters were compared with parameters of the HVF tests for each eye.

According to the results of a pilot study done on 30 eyes, the Pearson’s correlation coefficient (r) between average macular thickness and MD was calculated at 0.27. With an assumption of α = 0.05, power of 80%, and P ≤ 0.05, the minimum calculated sample size was 104 eyes. Statistical analysis was performed using SPSS software for Microsoft Windows version 16.0 (Chicago, IL, USA). A normal distribution of numeric data was confirmed using the Kolmogorov–Smirnov test. Pearson correlation’s coefficient (r) and linear regression analysis were used. A P-value ≤ 0.05 was considered statistically significant.

## RESULTS

The mean age of patients was 59.96 ± 8.75 years (range, 44–76 years) at the time of enrollment. The diagnosis of glaucoma was as follows: primary open angle in 77 eyes and pseudoexfoliation in 27 eyes. The clinical characteristics of the study eyes are summarized in Table 1.

Significant correlations were found between the cup-to-disc ratio and the AMT (r = -0.33, P = 0.001), MD-SWAP (r = -0.29, P = 0.003), MD-SAP (r = -0.43, P < 0.001), and PSD-SAP (r = 0.22, P = 0.02). In contrast, the correlation between the cup-to-disc ratio and the PSD-SWAP was not statistically significant (r = 0.13, P = 0.20). AMT correlated significantly with all measures of visual field, including MD-SWAP (r = 0.42), MD-SAP (r = 0.41), PSD-SWAP (r = -0.23), and PSD-SAP (r = -0.21), with P-values <0.001 for all correlations. Visual field measurements are described and compared by AMT in Table 2. The mean MD scores (using both SWAP and SAP) were significantly higher in the eyes with thin AMT compared to those with intermediate AMT (post-hoc Tukey’s test = 0.04 and 0.03, respectively). Other comparisons did not reach statistically significant levels.

**Table 1:** Clinical Characteristics of the 104 Study Eyes

Characteristic	Mean ± standard deviation (range)
Cup to disc ratio	0.60 ± 0.07 (0.40 to 0.70)
Average macular thickness (µm)	276.96 ± 7.80 (262.00 to 298.00)
Intereye difference	6.88 ± 5.43 (1.00 to 16.00)
Inferior macular thickness (µm)	274.82 ± 9.05 (260.00 to 300.00)
Intereye difference	7.35 ± 7.36 (1.00 to 21.00)
Superior macular thickness (µm)	276.89 ± 7.97 (263.00 to 298.00)
Intereye difference	5.29 ± 5.00 (0.00 to 17.00)
Superior macular thickness - inferior macular thickness (µm)	4.21 ± 4.77 (0.00 to 16.00)
Mean deviation-SWAP (dB)	-4.35 ± 2.00 (-9.38 to 5.80)
Intereye difference	1.16 ± 1.35 (0.06 to 6.27)
Mean deviation-SAP (dB)	-3.28 ± 1.36 (-5.89 to 1.20)
Intereye difference	0.93 ± 0.96 (0.07 to 4.09)
Pattern standard deviation-SWAP (dB)	3.31 ± 0.98 (1.79 to 6.50)
Intereye difference	0.87 ± 0.91 (0.04 to 2.80)
Pattern standard deviation-SAP (dB)	2.56 ± 0.61 (1.05 to 5.10)
Intereye difference	0.56 ± 0.65 (0.01 to 2.10)

SAP: Standard Achromatic Automated Perimetry

SWAP: Shortwave Automated Perimetry

µm: Micrometer

dB: Decibel

**Table 2:** Visual Field Measurements by Average Macular Thickness

Variables	Average macular thickness			P-value
	**Thin (20 eyes)**	**Intermediate (53 eye)**	**Thick (31 eyes)**	
MD-SWAP	-5.21 ± 0.63	-3.51 ± 1.40	-3.21 ± 0.33	0.04*
MD-SAP	-4.35 ± 0.21	-2.38 ± 1.21	-2.21 ± 0.11	0.02*
PSD-SWAP	4.90 ± 0.23	3.09 ± 0.70	2.67 ± 0.30	0.93
PSD-SAP	3.80 ± 0.70	2.39 ± 0.62	2.23 ± 0.27	0.80

Data are presented as mean ± standard deviation.

MD-SWAP: Mean Deviation Shortwave Automated Perimetry

MD-SAP: Mean Deviation Standard Automated Perimetry

PSD-SWAP: Pattern Standard Deviation Shortwave Automated Perimetry

(*) A P-value ≤0.05 was considered statistically significant.

**Figure 1:**
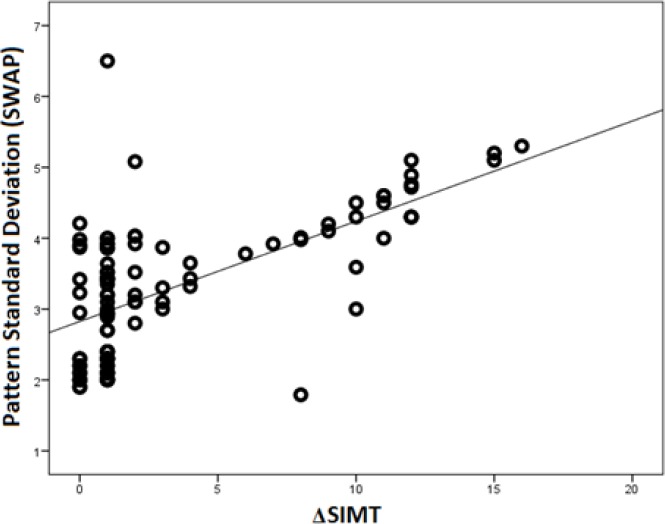
Scatterplot Representing the Correlation between the Difference in the Average Thickness of the Superior Macula and Inferior Macula (Δsimt) and Pattern Standard Deviation Measured By Shortwave Automated Perimetry (Swap)

**Figure 2:**
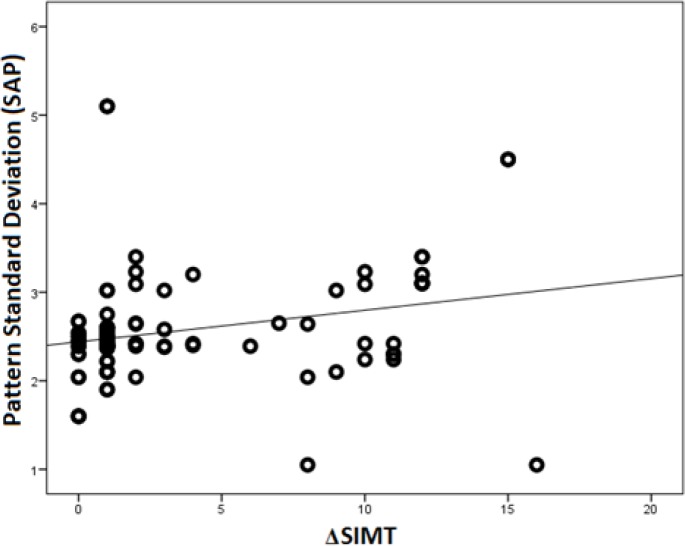
Scatterplot Representing the Correlation between the Difference in the Average Thickness of the Superior Macula and Inferior Macula (Δsimt) and Pattern Standard Deviation Measured By Standard Automated Perimetry (Sap)

The difference in the average thickness of the superior macula and inferior macula (ΔSIMT) correlated significantly with both PSD-SWAP (r = 0.63, P < 0.001, Fig 1) and PSD-SAP (r = 0.26, P = 0.01, Fig 2).

## DISCUSSION

In the present study, we showed a significant structure–function correlation between macular thickness measured by SD-OCT and visual field variables including SWAP and SAP. According to these findings, it could be concluded that macular thickness can help in confirming the presence and extent of visual field defects in patients with glaucoma. Previously, it has been suggested that demonstrating a structure–function relationship between retinal thickness and visual field parameters is a reliable, objective indicator of glaucoma, particularly in early stages of the disease when visual field testing is not applicable, and when its findings cannot be relied on. In addition, when glaucoma is suspected based on the appearance of the optic nerve only, macular thickness measurements could be used to devise an appropriate therapeutic plan tailored to that specific case [14]. Since the development of SD-OCT, dramatic advancements have been achieved in imaging of the macular region. This technique has accelerated image acquisition with higher resolution, allowing larger areas of the macula to be covered [24]. In addition, a high intervisit reproducibility of the SD-OCT has been reported, which is very useful in monitoring disease progression and the course of treatment [25].

Our findings are in line with several previous reports. Bagga et al. [26] reported a significant association between glaucomatous hemifield defects and decreased macular thickness. In another study by Kanadani et al. [27], abnormalities in macular thickness were detected using Stratus OCT with the fast macular thickness protocol. In the present study, a significant correlation between macular thickness abnormalities and glaucomatous functional defects was confirmed using HVF. Cho et al. [10] found SD-OCT useful to examine the strength and pattern of structure–function relationships between macular RGC complex thickness and visual field in superior and inferior quadrants. Despite reporting significant correlations, they suggested that using more extensively divided quadrants might have led into stronger relationships. In a study by Nakatani et al. [28], a significant association was found between SD-OCT-defined macular thickness abnormalities using a 6-mm grid and visual field MD. Using 10-2 HVF, Hood et al. [29] also found a direct, significant correlation between structural and functional defects in patients with glaucoma. In another study by that group [30], probability maps derived from OCT and visual field data revealed a significant association between structural and functional measures of glaucomatous damages. More recently, Boling et al. [5] tested a predictable structural relationship between macular thickness parameters and anatomically related visual field defects. To achieve this purpose, OCT macular scans and automated visual field of 127 eyes belonging to glaucoma patients were retrospectively examined. On the basis of their findings, significant associations were reported between each macular parameter and its anatomically related visual field defect. They suggested that macular scan OCT could be employed for diagnosis and management of glaucoma. Finally, in a similar retrospective work by Mathers et al. [14], high-resolution SD-OCT findings of a large area of the macula (8-mm grid) and HVF-derived MD and PSD scores were correlated in 73 patients with glaucoma. According to their findings, macular thickness correlated with HVF deficits, and much worse MD and PSD scores were found in the eyes with thinner macula (i.e., <270 µm) compared to those with thicker macula (i.e., >300 µm). The authors finally concluded that SD-OCT measurements of macular thickness correlate with HVF parameters in patients with glaucoma as well as glaucoma suspects, and this correlation is useful in confirming the existence and extent of the visual field defect. Of note and in conformity with the latter study, we also found that MD and PSD scores were worse in patients with thinner macula (<270 µm). Although glaucoma is typically a bilateral eye disease, it is usually asymmetric. A different involvement of the superior and inferior visual field is a hallmark of glaucoma. In addition, visual field defects are also frequently asymmetric between the two eyes at the time of diagnosis [31]. Therefore, using SD-OCT macular thickness maps that compare both intraeye asymmetry (between the superior macula and the inferior macula) and intereye asymmetry (between the two eyes) can help in the diagnosis and surveillance of the disease [32, 33]. In the present study, we also showed significant associations between both intraeye and intereye asymmetries with PSD.

In line with our findings, Mathers et al. [14] showed that an intraeye asymmetry of retinal thickness, including the superior or inferior macula, correlated directly with PSD. At the same time, they also showed a significant association of intereye asymmetry with visual field defects. These findings have been claimed clinically important, because a demonstration of asymmetry on OCT may increase the patient’s understanding of the severity of glaucoma and as a result, may lead to an increase in therapeutic compliance. Using a superior-to-inferior retinal thickness ratio in a 3-mm area of the macula located temporal to the fovea, Sihota et al. [34] also reported a significant association between asymmetry in retinal thickness and visual field defects. Both MD and PSD scores of the HVF have been found appropriate variables in monitoring glaucoma progression over time [14]. Available reports comparing the sensitivity of SWAP and SAP, however, are still inconclusive [35, 36]. To obviate this shortcoming, we used MD and PSD values obtained from both SWAP and SAP in the current study for the first time in the literature.

This study bears two limitations that need to be acknowledged here. First, excluding patients with vitreo-retinal pathology, with OCT segmentation artifact, or with unreliable visual fields may raise concerns about the generalizability. Second, using global measures of visual field defects including MD and PSD scores may underestimate the association between the visual field and the macular thickness. Using individual numerical data points of the HVF in future studies is recommended [37].

In conclusion, the present study showed a structure–function relationship in patients with glaucoma using SD-OCT determined macular thickness and visual field parameters. This association may help glaucoma specialists use macular thickness measurements for accurate and early diagnosis of glaucoma in suspected cases.
